# Adventitious Virus Detection in Cells by High-Throughput Sequencing of Newly Synthesized RNAs: Unambiguous Differentiation of Cell Infection from Carryover of Viral Nucleic Acids

**DOI:** 10.1128/mSphere.00298-19

**Published:** 2019-06-05

**Authors:** Justine Cheval, Erika Muth, Gaëlle Gonzalez, Muriel Coulpier, Pascale Beurdeley, Stéphane Cruveiller, Marc Eloit

**Affiliations:** a PathoQuest, Paris, France; b UMR1161 Virologie, ANSES, INRA, Ecole Nationale Vétérinaire d’Alfort, Université Paris-12 Est, Maisons-Alfort, Paris, France; c Ecole Nationale Vétérinaire d’Alfort, Université Paris-Est, Maisons-Alfort, Paris, France; d Pathogen Discovery Laboratory, Biology of Infection Unit, Institut Pasteur, Paris, France; University of Maryland, College Park

**Keywords:** 4-thiouridine, RNA-seq, Vero, biologicals, high-throughput sequencing, metagenomics, squirrel monkey retrovirus, tick-borne encephalitis virus, virus safety

## Abstract

The use of high-throughput sequencing (HTS) to identify viruses in biologicals differs from current molecular approaches, since its use enables an unbiased approach to detection without the need to design specific primers to preamplify target sequences. Its broad range of detection and analytical sensitivity make it an important tool to ensure that biologicals are free from adventitious viruses. Similar to other molecular methods, however, identification of viral sequences in cells by HTS does not prove viral infection, since this could reflect carryover of inert viral sequences from reagents or other sources or the presence of transcriptionally inactive cellular sequences. Due to the broad range of detection associated with HTS, the above can potentially be perceived as a drawback for the testing of pharmaceutical biological products using this method. In order to avoid the identification of inert viral sequences, we present a methodology based on metabolic RNA labeling and sequencing, which enables the specific identification of newly synthesized viral RNAs in infected cells, resulting in the ability to unambiguously distinguish active infection by DNA or RNA viruses from inert nucleic acids. In the present study, we report the ability to differentiate Vero cells acutely infected by a single-stranded positive-sense RNA virus (tick-borne encephalitis virus) from cells which have been in contact with nonreplicating virus particles. Additionally, we also found a laboratory contamination by the squirrel monkey retrovirus of our Vero cell line, which was derived from an Old World (African green) monkey, a type of contamination which until now has been identified only in cells derived from primates from the New World.

**IMPORTANCE** The use of high-throughput sequencing (HTS) to identify viral contamination of biological products is extremely sensitive and provides a broad range of detection. Nevertheless, viral sequences identified can also be inert. Examples include contamination resulting from reagents or the presence of inactivated viruses in animal-derived components of the cell culture medium. We therefore developed a method that relies on the sequencing of newly synthesized RNAs, an unequivocal sign of the presence of a transcriptionally active virus. This improvement in the specificity of viral testing increases the acceptability of HTS as a standard test for cells used in manufacturing biologicals and in biotherapies.

## Abstract (precis)

The use of high-throughput sequencing (HTS) to identify viral contamination of biological products is extremely sensitive and provides a broad range of detection. Nevertheless, viral sequences identified can also be inert. Examples include contamination resulting from reagents or the presence of inactivated viruses in animal-derived components of the cell culture medium. We therefore developed a method that relies on the sequencing of newly synthesized RNAs, an unequivocal sign of the presence of a transcriptionally active virus. This improvement in the specificity of viral testing increases the acceptability of HTS as a standard test for cells used in manufacturing biologicals and in biotherapies.

## INTRODUCTION

Viral contamination is a topic of major concern for biological products. This includes both the risk of contamination of good manufacturing practice (GMP) facilities and the final drug product. Virus testing of raw materials, cells, and virus seeds is key for the safety of the drug product. This is particularly critical for live vaccines, gene therapy viral vectors, and cell therapy drug products, since their production does not include downstream adventitious virus elimination steps. As a result, the safety of these products relies heavily on viral testing during the production process. All previously reported contaminations of products based on cell cultures were due to unexpected animal viruses that remained unidentified despite viral testing of raw materials or production cells. The details of these contaminations have been reviewed by others (1). In fact, classical viral testing is limited, since many viruses do not grow in cell lines used for *in vitro* tests or in rodents or eggs used for *in vivo* tests (2). While targeted PCR represents a potential alternative approach, it targets only a small range of known viruses and it lacks the ability to identify the ever-increasing number of newly discovered viruses or unknown viruses.

With the above in mind, high-throughput sequencing (HTS)-based viral testing methods are particularly appealing due to their unbiased and wide viral detection range which includes unknown viruses. This is particularly important, as the catalog of animal viruses is still incomplete, an intrinsic limit to any targeted testing method. HTS is a nucleic acid-based technology that makes no *a priori* assumptions regarding viruses to be detected, since it identifies all viral nucleic acid sequences found in a sample. Similar to other molecular methods like PCR, the identification of viral sequences in cells by HTS does not prove viral infection, since many other sources of inert sequences exist. These inert sequences can be exogenous sequences, an example being bovine virus from fetal bovine serum (FBS) even after it has been properly inactivated by gamma irradiation. These sequences can be found associated with cells, because inactivated viruses can still bind to cells. Other sources of inert sequences are present in reagents and can contaminate a sample during the nucleic acid extraction procedure or subsequent steps (3–5).

While the discovery of inert viruses is not an issue for targeted PCR, because this method is limited to a few viruses, it represents a major problem when using HTS, as this method has a much broader range of viral detection, a main advantage of the technology. As a result, HTS can potentially identify inert viral sequences leading to unnecessary, lengthy, and resource-consuming investigations to rule out or confirm cellular infection.

In order to distinguish the identification of inert sequences versus active cell infection, we decided to focus HTS solely on real markers of cell infection for the present study, specifically the synthesis of RNAs that encode viral proteins and/or regulate the expression of other genes. We have recently demonstrated that sequencing positive-sense or negative-sense cellular viral RNAs that differ from genomic nucleic acids can selectively pinpoint productive or latent viral cell infection (6). In the present paper, we present a streamlined RNA sequencing (RNA-Seq) pipeline based on metabolic RNA labeling which enables specific identification of RNAs synthesized in cells and provides unambiguous evidence of active cellular infection versus inert nucleic acids. We report the results corresponding to Vero cells acutely infected by a positive-sense single-stranded RNA virus (tick-borne encephalitis virus [TBEV]) and the identification of the unexpected laboratory persistent contamination of the cells by squirrel monkey retrovirus (SMRV).

## RESULTS

### Identification of adventitious viruses by agnostic RNA-Seq in Vero cells.

Vero cells were first put in contact with a high dose of tick-borne encephalitis virus (TBEV) at +4°C (day 0 [D0]). At this temperature, only passive virus binding to cell receptors occurs, and virus entry, an active event, is blocked. Therefore, this experimental setting mimics the carryover of a nonreplicating virus. RNAs were extracted and sequenced as a marker of DNA or RNA virus infection. The results of the agnostic analysis and those of the mapping of the reads against the two main viral hits found by the agnostic analysis (TBEV and squirrel monkey retrovirus [SMRV]) are shown, respectively, in Table S1 in the supplemental material and in Table 1. The main viral species detected at D0 was, as expected, TBEV, but also, unexpectedly, SMRV (Table 1). More than 160,000 TBEV reads out of a total of around 150 million raw reads (Table S1) were identified, covering the whole genome. Vero cells were then shifted to 37°C to enable virus entry and then incubated for 1 day prior to harvest. The number of reads strongly increased with between 5.2 million and 6.4 million TBEV reads recorded. Additionally, between 1.6 and 1.8 million reads mapping to SMRV-H (an SMRV isolated from a human lymphoid cell line [7]) were also identified independent of the day of harvest. Taking these results together, this means that the SMRV transcripts were expressed by the cells without any relationship to experimental infection by TBEV.

**TABLE 1:** Number of reads on TBEV and SMRV genomes and horizontal coverage of the genome^*a*^

Virus and parameter	Value for parameter at the following time and treatment			
	Day 0, no 4sU	Day 1, no 4sU	Day 1, 4sU + alkylation	Day 1, 4sU, no alkylation
TBEV				
No. of reads^*b*^	160,085 (0.27)	6,408,291 (0.35)	5,240,211 (0.36)	5,722,338 (0.32)
Horizontal coverage (%)^*c*^	100	100	100	100

SMRV				
No. of reads	1,807,960 (0.39)	1,601,090 (0.40)	1,816,788 (0.48)	2,479,933 (0.37)
Horizontal coverage (%)	100	100	100	100

a Reads were mapped on the genomes of TBEV and SMRV found by the agnostic procedure (see Table S1 in the supplemental material).

b The number of reads shows the number of reads of the virus on the genome. The values in parentheses are the percentages of negative-sense/total number of reads.

c Horizontal coverage of the genome is shown as a percentage of the genome.

10.1128/mSphere.00298-19.2 TABLE S1 Agnostic analysis. The number of reads following each step of the filtering process and results of the *de novo* assembly and of the BLAST analysis are shown. Download Table S1, PDF file, 0.07 MB. Copyright © 2019 Cheval et al. 2019 Cheval et al. This content is distributed under the terms of the Creative Commons Attribution 4.0 International license.

We also identified a number of other hits (Table S1). The main additional hit was baboon endogenous virus, a known endogenous virus of Vero cells (8). We used the human genome as a reference (GRCh37/hg19) for depleting host sequences, which explains why this baboon endogenous virus was detected. A few hundred reads mapping to endogenous human retroviruses were also recorded. In our experience, this finding is frequent in primate/human cell lines. We also found a few bovine viral diarrhea virus (BVDV) reads typically associated with the use of gamma-irradiated bovine serum. In addition, we identified a few reads (<50) targeted to different herpesviruses which we considered background noise.

### Differentiation of cell infection versus carryover of inert sequences.

Since our primary objective was to mimic challenging conditions for differentiation between cell infection from carryover while testing the ability of HTS to detect early infection of cells, we compared results of cells put in contact with high doses of TBEV blocked for virus replication at +4°C with those of cells infected with the same dose of virus 24 h postinfection. The former mimicked cells with inactivated virus or free nucleic acids, and the latter mimicked cells infected just before being banked. Since TBEV is a positive-sense single-stranded RNA (ssRNA) virus, the negative-sense RNA was used as a marker of virus replication. The three conditions tested at D1 resulted in 0.32% to 0.36% of the reads being negative sense compared to 0.27% at D0, a very small but highly significant difference (*P* < 0.0001 by chi-square test) (6). This type of comparative analysis is not relevant for the chronic infection of cells by SMRV, a retrovirus for which transcription uses a DNA provirus as the matrix and leads mainly to positive- but also to negative-sense RNAs (9).

**FIG 1:**
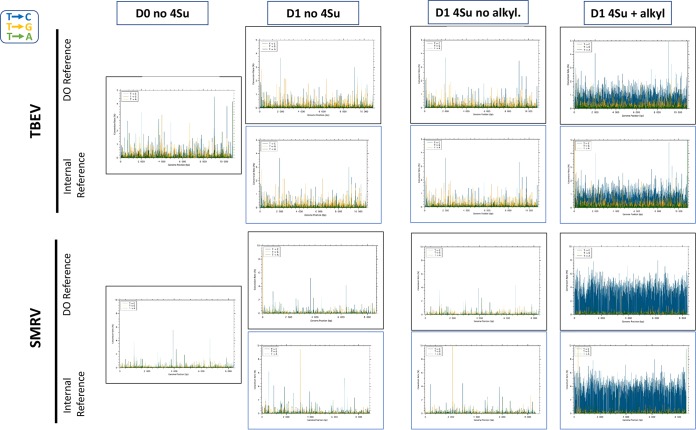
Distribution of conversion rates along the genomes of SMRV and TBEV. Conversion rates were calculated in reference to the reference sequence derived from day 0 (“TBEV REFERENCE” and “SMRV REFERENCE”) or from an internal consensus sequence based on the reads from each experimental condition (“INTERNAL REFFERENCE”). For the D0 time point, the two modes of calculation are therefore equivalent. (T to C, G, or A, in blue, orange, or green lines, respectively). Abbreviations: D0 and DO, day 0; 4Su, 4-thiouridine; alkyl, alkylation.

**FIG 2:**
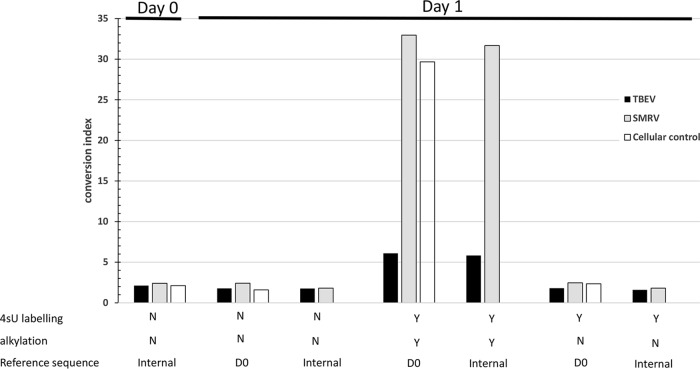
Conversion indexes expressed as the ratio of the T→C conversion rate to the average of the (T→A, T→G) conversion rates. Conversion rates for viruses were calculated according to the two types of reference sequence as in Fig. 1. Conversion rates for cellular RNA controls are calculated according the CONTROL REFERENCE sequence. Abbreviations: N, no; Y, yes.

We then examined the TBEV rate of T→C conversion and their distribution along the whole viral genomes following metabolic labeling by 4-thiouridine (4sU) of newly synthesized RNAs (Table 2 and Fig. 1 and 2). At D1 and in the absence of metabolic labeling, the T→C rate was very low (0.16%) and similar to those of T→A or T→A (0.05 to 0.13%), resulting in a calculated background conversion index of 1.78. Similar results were obtained at D0, indicating good reproducibility of the background level of conversion. These background rates of T conversion were distributed along the whole genomes (Fig. 1). In clear contrast, the T→C conversion rate for labeled and alkylated RNAs at D1 was much higher (0.87%), resulting in a conversion index of 6.09, a 3.4-fold increase compared to the background at D1. The conversion index at D1 for the labeled and alkylated SMRV-infected cells was 32.95, 13.6-fold over the background level. These specific T→C conversion rates were also distributed along the whole viral genomes (Fig. 1), supporting the use of the mean conversion rates as a robust indicator of efficacy of labeling.

**TABLE 2:** Conversion rate of T nucleotides and conversion index

Sample and parameter	Value for parameter for sample at the following time and treatment^*a*^:			
	Day 0, no 4sU	Day 1, no 4sU	Day 1, 4sU + alkylation	Day 1, 4sU, no alkylation
TBEV				
T→C rate (%)	0.15	0.16/0.16	0.87/0.82	0.16/0.14
T→A rate (%)	0.05	0.05/0.05	0.10/0.10	0.05/0.05
T→G (%)	0.11	0.13/0.13	0.18/0.18	0.12/0.12
Conversion index	2.12	1.78/1.76	6.09/5.83	1.81/1.60

SMRV				
T→C rate (%)	0.15	0.15/0.11	3.49/3.45	0.15/0.11
T→A rate (%)	0.05	0.05/0.05	0.10/0.10	0.05/0.05
T→G rate (%)	0.07	0.08/0.08	0.11/0.12	0.07/0.08
Conversion index	2.41	2.42/1.80	32.95/31.67	2.46/1.80

Cellular transcripts (internal control)				
T→C rate (%)	0.10	0.06	1.83	0.05
T→A rate (%)	0.03	0.02	0.04	0.01
T→G rate (%)	0.06	0.05	0.08	0.03
Conversion index	2.12	1.60	29.67	2.34

a In each TBEV and SMRV cell, the first value (before the slash) and the second value (after the slash) correspond to the conversion rate or index based on the external consensus sequence derived from the D0 reads (“TBEV REFERENCE” and “SMRV REFERENCE”) or from internal consensus sequences directly derived from the reads of the experimental condition (“INTERNAL REFERENCE”), respectively.

Comparisons between metabolically labeled and nonlabeled RNAs would necessitate two conditions of culture. As a result, we also compared the TBEV and SMRV conversion indexes obtained at D1 for the 4sU-labeled culture, with and without RNA alkylation. This necessitates only one condition of culture, followed by RNA extraction and alkylation, or no treatment. The low level of conversions in cells with 4sU-labeled, nonalkylated RNA did not impair the detection by BLAST analysis of potential viral hits (Table S1). As shown in Table 2, the conversion index of the 4sU-labeled, nonalkylated RNAs remained low and close to that of the nonlabeled condition (1.81 and 2.46 for TBEV and SMRV, respectively, increasing 3.4- and 13.4-fold, respectively, in the alkylated condition). This suggests that nonalkylated RNAs extracted from the same cell culture can be used to calculate background conversion rates.

We have also taken advantage of the fact that even in the best cases, the rate of T→C conversion was low (below 5% of T positions) and therefore should not modify the consensus sequence, which represents the most frequently found base for each nucleotide position. For SMRV and TBEV, we recalculated all conversion rates and their distribution along each genome (Fig. 1 and Table 2). As expected, this did not significantly modify the results obtained when the nonlabeled RNA sequence (D0 reference) was used. The only conversion indexes above the background level were those of 4sU-labeled and alkylated RNAs of TBEV- and SMRV-infected cells at D1. The conversion indexes at D1 were 3.3- and 17.6-fold over the background of nonlabeled cells for TBEV and SMRV, respectively.

Therefore, our results show that after 4Su labeling of cells, RNA-Seq was able to specifically identify newly synthesized viral RNAs with a high signal-to-background noise ratio in a one-shot test, without the need for an external reference sequence.

### Characterization of expression of SMRV sequences in Vero cells.

With the reads mapping to SMRV-H, we derived a consensus full-length sequence, deposited in GenBank (10) as proviral DNA under the name SMRV-Vero (GenBank accession number MK561030). The full DNA genome has a length of 8,787 nucleotides (nt) and shows 99% identity with SMRV-H. Compared to the original SMRV isolated from squirrel monkey lungs, SMRV-Vero, like SMRV-H, shows an insertion of one (SMRV-Vero) or two (SMRV-H) nucleotide G in the primer binding site (PBS) (at nt 467 in SMRV-H). The four open reading frames (ORFs) described for SMRV-H are also present as shown in Table 3. Amino acid identity ranges from 98% to 99% for all proteins except for Gag (93%). The Gag precursor protein is cleaved into p10, p16, p19, and p35 proteins. Most of the variability (Fig. S1) was concentrated in the p35 protein, the major capsid protein (11).

**TABLE 3:** Comparison of SMRV-H and SMRV-Vero proteins

Protein	Length of protein (no. of aa) in:		Amino acid identity (%)
	SMRV-H	SMRV-V	
Gag	740	742	93
Protease	90	90	98
Pol	798	798	99
Env	575	575	98

10.1128/mSphere.00298-19.1 FIG S1 Alignment of SMRV-H and SMRV-Vero Gag precursor proteins. Download FIG S1, PDF file, 0.08 MB. Copyright © 2019 Cheval et al. 2019 Cheval et al. This content is distributed under the terms of the Creative Commons Attribution 4.0 International license.

### RT-PCR investigations.

Reverse transcription-PCRs (RT-PCRs) targeting the *gag* and *env* gene together with the primer binding site confirmed the presence of SMRV expression in the Vero cell line. Sanger sequencing confirmed the sequence of the PBS at position nt 467 (SMRV-H position). RT-PCRs were negative for the same lot of cells that was banked 3 passages after receiving the cells from ATCC and also for a vial of another lot of Vero cells (lot 70005907) tested directly without any cultivation step. This demonstrated that contamination occurred during the passage of cells in the cell culture laboratory.

## DISCUSSION

The efficacy of HTS for the testing of biologicals has been demonstrated by the confirmation of viral contamination of a marketed live vaccine despite no findings by conventional testing (12). The potential expanded use of HTS for testing of biologicals, however, would need to address the concern of finding viral hits that do not present safety concerns. Our objective was to design HTS strategies able to add specificity of detection of living viruses in cells in addition to the broad range of viral detection and analytical sensitivity that this technology provides versus current testing methodologies. We have previously demonstrated that our protocol of deep sequencing of RNAs is sensitive enough to detect a single human herpesvirus type 4 (HHV-4)-infected cell in a background of 10^5^ virus-free cells and also has the ability to detect cells infected at very low multiplicity of infection (MOI) by BVDV 2 days after infection (6). Coupled with strand analysis as biomarkers of virus expression during replication and even latency (13, 14), this protocol increases specificity compared to sequencing all types of cellular nucleic acids (DNA and RNA) (6). Nevertheless, this method is not devoid of limits. For example, since antigenomes are present in small amount for single-stranded positive-sense RNA [ss(+)RNA] viruses, very deep sequencing is required. For some single-stranded negative-sense RNA [ss(−)RNA] viruses, full-length antigenomes are also present in mature virions, which results in the requirement for an additional, virus species-specific bioinformatic analysis in order to identify subgenomic transcripts associated with cell infection. Cell-free viral RNAs can also be present in standard virus stocks frozen at −80°C or in liquid nitrogen just after harvest and therefore can introduce background noise during validation without any relevance to real samples.

As a result of the above, we decided to evaluate an ultimate evolution of the concept of targeting RNAs specifically for viral infection, the identification of newly synthesized RNAs, since this could distinguish viral expression from carryover, independently of the type of virus. A classical method for such analysis would be to label nascent RNAs with modified uridine analogs (e.g., 4-thiouridine [4sU], 5-etyniluridine [EU], and 5′-bromouridine [BrU]), followed by purification of labeled *de novo* RNAs (see the review in reference 15). To draw any conclusion regarding carryover of inert viruses versus cell infection, these techniques would have necessitated comparing the distribution of viral hits between purified RNAs and crude RNAs, which would be difficult to standardize and validate. For the present study, we have taken advantage of a recently described thiol (SH)-linked alkylation for the metabolic sequencing of RNA (SLAM seq), which enables 4sU detection in RNA species after metabolic RNA labeling (16).

After performing metabolic RNA labeling for 6 h, we prepared total RNA followed by thiolalkylation or no treatment and then randomly deep sequenced the libraries. We based our interpretation on the frequency of the T→C conversion compared to the background of T→G and T→A. Using the ss(+)RNA virus TBEV as a model, we demonstrated the ability to differentiate “old” RNAs of nonreplicating viruses bound at the cell surface from RNAs associated with live viruses. This is an obvious advantage compared to the detection of whole nucleic acids, since these are not specific to live viruses. This method also overcome the difficulty of detecting the small amount of antigenomic RNA strand as a marker of ss(+)virus expression (6). Another advantage of our technique is that only RNA is labeled. This is an interesting feature since RNAs are *per se* evidence of viral transcription for DNA viruses and retroviruses (which are transcribed from a DNA provirus). Even some transcripts incorporated in inactivated virions of some enveloped double-stranded DNA (dsDNA) viruses, such as herpesviruses (17, 18), poxvirus (19), or mimivirus (20), would not be labeled.

A good demonstration of the advantages of our nontargeted approach was that we were able to detect the expression of a strain of SMRV very close to SMRV-H in the Vero ATCC CCL81 cell line used for our TBEV infection studies. We have named this strain SMRV-Vero. When tested *a posteriori* by RT-PCR, it appeared that the Vero cells from ATCC were SMRV free and that the contamination arose after receiving the cells from ATCC in the laboratory in which the cells were cultivated, from a currently unknown origin. This was unexpected, as SMRV, a type D betaretrovirus, is known to infect only primates from the New World (21, 22), and Vero cells are derived from African green monkeys, an Old World primate. Until now, only different simian endogenous retroviruses (SERVs) have been identified in Vero cells (8, 12, 23, 24). This includes one type close to baboon endogenous virus (BAEV) gammaretrovirus (25) and another close to exogenous simian retrovirus (SRV) (26). These sequences, which include complete proviruses (27), show a large diversity in the cell origin, but most harbor inactivating deletions or frameshift mutations (ATCC CCL81, JCRBO111, or Vero 76) (24, 27). The SMRV-Vero strain that we identified does not present any obvious loss-of-function mutations, insertions, or deletions. Compared to the SMRV strain isolated from monkeys in canine cells, the G insertion in the PBS of SMRV-Vero is reminiscent of the GG insertion found in SMRV-H, a clone persistently infecting human lymphoid cell lines and should therefore be compatible with priming of reverse transcription by tRNALys1,2. Mutations with reference to SMRV-H were identified in the p35 capsid protein. It is currently not known whether SMRV-Vero produces an infectious virus. More characterization of this virus is currently ongoing.

Regarding the testing of cells (e.g., cell banks, drug products in cell therapy, “control cells” tested during vaccine production, cells used for bioassay of raw materials), routine usage of our technique requires only the addition of 4sU to the cell medium a few hours before cell harvest.

In a first modality of usage, agnostic HTS analysis is completed using the sequences obtained with an index sample (nonalkylated RNA from the 4sU-labeled cells or nonlabeled cells). The use of nonalkylated RNA from the 4sU-labeled cells is manageable, since alkylation can be performed as a second step, allowing comparison of sequences derived from libraries with the same contents, and because such comparison does not necessitate a parallel, nonlabeled culture. A consensus reference sequence has to be derived from 4sU-labeled, nonalkylated RNAs. Conversion rates and conversion ratios then have to be calculated by mapping the reads derived from the 4sU-labeled and alkylated RNAs onto this reference sequence. Our results suggest that if nonlabeled cells are used as a reference, an increase of at least threefold between the conversion indexes could be used to distinguish newly synthesized RNAs. We found that the same threshold could be used in our study when RNA from 4sU-labeled, nonalkylated RNAs was used as a reference. This is despite reports of a significant T→C conversion rate in 4sU-labeled nonalkylated RNAs (16).

In a second and privileged modality of usage, the protocol uses only one set of sequences from the tested cell sample. Specifically, 4sU-labeled and alkylated RNAs would be sequenced and analyzed by the agnostic pipeline. The performance of the agnostic pipeline is not significantly modified (Table 1), as only a minority of T positions are converted to C. In case of any viral hits, the targeted pipeline will use the same set of sequences to first define a consensus sequence, itself marginally impacted by the low rate of T→C conversions, and then to calculate the conversion rate and index. In this setting, the conversion index will be used to determine whether the viral hit corresponds to an RNA that has been recently transcribed. Our results suggest that a conversion index above 3 would provide a good margin of safety above the background. Proper optimization of 4sU labeling conditions and threshold determination with the cells to be tested could improve the quality of the results and are mandatory in the case of GMP routine testing.

The protocol needs to compute statistics and the frequency of T conversions and is not useful when a few reads are evidenced (for example, see BVDV in Table S1 in the supplemental material). In practice, such a low number of reads can hardly be mistaken with a viral infection, as shown when using worst-case models (6).

In conclusion, using transcriptome analysis by HTS coupled with RNA strand analysis and identification of newly synthesized RNAs after metabolic labeling enables the ability to broadly and specifically test cells for active infection versus inert viral sequence contamination, thus representing technical progress for the viral safety testing of biological products.

## MATERIALS AND METHODS

### Cells and viruses.

A vial of Vero cells (ATCC-CCL-81; lot 62488537; ATCC, Molsheim, France) was cultivated in minimum essential medium (MEM) supplemented with 10% fetal bovine serum (FBS) after receiving the cells and frozen at passage 3 (the first passage of the vial of cells received from ATCC was designated passage 1). A vial was then defrosted in a biosafety level 3 (BSL-3) laboratory to be used as cell substrate for virus cultivation, and the cells were grown again in MEM supplemented with 10% FBS. Cells were used at passage 18. A second vial of Vero cells (lot 70005907) was obtained from the same source and used directly for PCR testing. Tick-borne encephalitis virus (TBEV), a member of the family *Flaviviridae*, possesses a single-stranded RNA with positive polarity [ssRNA(+)] genome. The Hypr strain (28) was kindly supplied by Sarah Moutailler, ANSES, Maisons-Alfort, France.

### TBEV infection of Vero cells.

Vero cells were plated at 400,000 cells/well in three wells of a six-well culture plate (9.5 cm^2^ [cell growth area]) in order to reach 10^6^ cells/well after 24 h. Cells were then infected with the Hypr strain TBEV at a multiplicity of infection of 1 and incubated for 1 h on ice with agitation. The medium was removed in one well just after incubation, and the cells were lysed with 1 ml of TRIzol and stored at −80°C until RNA extraction (condition 1, day 0 with no 4sU [“D0 no 4sU”]). For the other two wells (conditions 2, 3, and 4, see next paragraph), the medium was removed and replaced by MEM plus 10% FBS and incubated overnight at 37°C.

### 4sU labeling and RNA extraction.

The addition of 4-thiouridine (4sU) into the cell culture medium enables 4sU nucleotides to be incorporated into newly synthesized RNA. The reverse transcription of 4sU displays a certain percentage of misincorporation resulting in a T→C transition in the cDNA that can be identified by sequencing (16). Media containing 800 μM 4sU was prepared by adding 8 μl of 100 mM 4sU in 992 μl of MEM. The day after viral infection (day 1 [D1]), the medium was removed and replaced by medium without 4sU in one well (condition 2, day 1 with no 4sU [“D1 - no 4sU”]) or 4sU-containing medium (800 μM) for the other well (conditions 3 and 4, day 1 with 4sU [“D1 with 4sU”]). Six hours later, the medium was removed and replaced by fresh medium without 4sU in the condition “D1 - no 4sU” or by fresh 4sU-containing medium (800 μM) in the two “D1 with 4sU” conditions. Three hours later, the medium was removed from the three wells, and the cells were lysed with 1 ml of TRIzol and stored at −80°C until RNA extraction. RNA extraction was performed in the dark using a chloroform-isoamyl alcohol mix (24:1) (catalog no. 25666; Sigma-Aldrich, St. Louis, MO, USA) followed by isopropanol-ethanol precipitation. During extraction, reducing agent was used to maintain the 4sU-treated sample under reducing conditions. Alkylation was performed using the SLAMseq kinetic kit with anabolic kinetics module (catalog no. 061; Lexogen, Vienna, Austria) for the “D1 with 4sU” condition. Total extracted RNA was mixed with iodoacetamide, which modifies the 4-thiol group of 4sU-containing nucleotides via the addition of a carboxyamidomethyl group, leading to condition 3, “D1 with 4sU+alkylation.” This alkylation amplifies the frequency of T→C misincorporations during reverse transcription. Condition 4 was labeled “D1 with 4sU no alkylation.” The RNA was then purified using ethanol precipitation before library preparation.

### Library preparation and sequencing.

The SMARTer Stranded Total RNA-Seq kit (Pico Input Mammalian) (ClonTech, Mountain View, USA) was used for direct construction of libraries starting with 10 ng of RNA. The workflow used with this kit incorporates a proprietary technology that depletes ribosomal cDNA using probes specific to mammalian rRNA and some mitochondrial RNA. Sequencing was performed on the NextSeq system (Illumina, San Diego, CA, USA) using the NextSeq 500/550 high output kit v2 (catalog no. FC-404-2002; Illumina). Single-read sequencing was utilized with a read length of 150 nucleotides, resulting in the generation of approximatively 125 million reads per sample.

### Agnostic bioinformatic analysis.

The raw data reads were filtered to select high-quality and relevant reads. Raw data were sorted to suppress or cut duplicates, low-quality reads, and homopolymers (proprietary software; PathoQuest, Paris, France). Sequences introduced during the preparation of Illumina libraries (adapters, primers) were removed with Skewer (29). Reads aligned with BWA (30) to (i) the human genome (reference GRCh37/hg19 [31]) used as a proxy to mimic primate (Vero cells) host background or to (ii) bacterial rRNA were discarded. The bacterial rRNA database was initially downloaded from the EMBL-EBI ENA rRNA database (ftp://ftp.ebi.ac.uk/pub/databases/ena/rRNA/release) and reworked using in-house sequence cleaning and clustering processes. Remaining reads were considered sequences of interest and were assembled into contigs with CLC Assembly Cell solution (Qiagen Bioinformatics). Resulting contigs and nonassembled reads (singletons) were aligned using BLAST alignment (32) on viral and comprehensive databases. Contigs and singletons were first aligned on a viral nucleotide database. Hits with an E value below 10^−3^ were aligned on a comprehensive nucleotide database. If the best hit was still a viral taxonomy, hits were reported. The nucleotide viral and comprehensive databases were downloaded during November 2017 from the EMBL-EBI nucleotide sequence database (https://www.ebi.ac.uk/). The viral database is restricted to viral sequences of the EMBL-EBI nucleotide sequence database, including subgenomic sequences. Proprietary software was developed to remove duplicate and low-confidence sequences (e.g., too short, multiple taxonomies, low-quality associated keywords). Contigs without any viral nucleotide hits were similarly aligned successively on viral and comprehensive protein databases to check for more-distant viral hits. Protein viral and comprehensive databases were downloaded during November 2017 from the Uniref100 database (https://www.uniprot.org). Though the Uniref100 database is already nonredundant, we performed a taxonomic cleaning process to generate the final databases. The taxonomic assignation reported the best hit results with contigs not assigned after these two rounds of alignment being classified as unknown or nonviral species.

The data from condition 1 (“DO no 4sU”) enabled the identification of contigs covering the whole genome of TBEV (*n* = 1; length = 10850 bp) and SMRV (*n* = 4; cumulated length = 8639 bp). For SMRV, a new assembly of the reads with Megahit v1.1.3 (33) gave a unique contig covering the whole genome. The resulting sequences, obtained at D0, were labeled “TBEV REFERENCE” and “SMRV REFERENCE.”

### Estimation of T→C conversion ratio.

In order to detect viral sequences with a very high number of T→C conversions, the set of quality filtered reads was mapped back to TBEV/SMRV REFERENCE with minimap2 (34). The pileup module of the htsbox software (https://github.com/lh3/htsbox) was then used to detect all mismatches (with a base quality at least equal to 30) at every position of the TBEV or SMRV REFERENCE. The global variation profile was then analyzed by a proprietary script (PathoQuest, Paris, France) to define each nucleotide conversion rate. The proportion of converted nucleotides was compared to the total number of aligned nucleotides. For example, the T→C conversion rate was calculated using the following formula:

$$\text{T}\to \text{C rate}=\frac{\text{No. of C nucleotides identified when a T was expected}}{\text{Total no. of expected T}}$$

The conversion rates for each time point were normalized with the following conversion index:

conversion index=T→C rateMean(T→A, T→G rates)

Additionally, for viral hits, conversion rates were also calculated using as reference the consensus sequence derived from the reads from each experimental condition. This consensus sequence was named “INTERNAL REFERENCE.” Hence, for the D0 time point, the “TBEV REFERENCE” and “SMRV REFERENCE” were equivalent to “INTERNAL REFERENCE” sequences.

As a quality control for the labeling, we checked the mean conversion index of a set of exons using nonlabeled cells as a reference. Exons were used from the following human genes (35) (RefSeq accession number): C1orf43 (NM_015449), CHMP2A (NM_014453), EMC7 (NM_020154), and GPI (NM_000175). We used these human exons in order to identify their equivalent in the Chlorocebus sabaeus genome, from where the Vero cells are derived. The complete assembly of *Chlorocebus sabaeus* (accession number GCF_000409795.2) was retrieved from NCBI assembly database (https://www.ncbi.nlm.nih.gov/assembly/). Selected human exons were mapped onto *C. sabaeus* assembly using minimap2 (34), and the resulting bam file was converted to a bed file using the bamtobed module from the BEDTools utility (36). Only hits with mapping quality higher than 30 were retained (41 exons), and the corresponding sequences were extracted from *C. sabaeus* assembly using the getfasta module from the BEDTools utility and indexed for analyses as the “CONTROL REFERENCE” sequence to calculate conversion rates and indexes. Labeling was considered satisfactory if the conversion index was superior to 10.

### Stranded analysis.

A targeted and stranded analysis was performed on the identified TBEV reads. This analysis was based on a more stringent mapping alignment of filtered reads with the alignment providing a detailed horizontal genome coverage and depth profile. Local alignments were performed with BWA (30). Since the sample libraries were prepared using the SMARTer Stranded RNA-Seq kit, the RNA strand information was also retained. As a result, a mapping alignment analysis was able to provide information on the mother strand (the DNA or RNA template sequence from which the RNA was transcribed) of each read (sense or reverse relative to the mother strand) as described previously (6).

### SMRV RT-PCR.

Primers used for RT-PCR included SMRV-gag-4F (ACCGTGTTTTTGGTCCTGAG), SMRV-gag-4R (GGGCACTGCTGTAGGAACAT), SMRV-PBS-F (CTGCGGGACAGAGCAAGT), SMRV-PBS-R (TCCCATGATTGGGTCTTACC), SMRV-env-4F (GTACAGCAGGACTCGGGGTA), and SMRV-env-4R (CGTCTTCGTTCGAGGTCTTC). cDNA was tested using 100 ng of RNA to be tested in a final volume of 10 μl with the SuperScript III First Strand Synthesis System kit (catalog no. 18080-051; Thermo Fisher, Waltham, USA) and then diluted to 2 ng/μl. From the RNA samples, 2 μl was used for each tube, and PCR was conducted with the PrimeSTAR GXL DNA polymerase kit (catalog no. R050A, Ozyme, Montigny-le-Bretonneux, France). Thirty cycles were performed, and each cycle consisted of 10 s at 98°C, 15 s at 60°C, and 1 min at 68°C.

### Data availability.

Access to the proprietary software tools used in this study will be granted to researchers upon request.
