# Supplementary material for: Adventitious Virus Detection in Cells by High-Throughput Sequencing of Newly Synthesized RNAs: Unambiguous Differentiation of Cell Infection from Carryover of Viral Nucleic Acids
Source: mSphere. 2019 Jun 5;4(3):e00298-19. doi: 10.1128/mSphere.00298-19 (PMC6553555; doi:10.1128/mSphere.00298-19)
Supplement: TABLE S1 [file mSphere.00298-19-st001.pdf]

	DO : no 4 SU	D1 : no 4sU	D1 : 4sU + alkylation	D1 : 4sU no alkylation
Total reads	153379022	153360490	149549706	163035957
**FILTERING**				
Duplicates/Quality	84575371	85885045	73631007	82102171
Adapters	84574497	85884068	73629933	82101241
Host	19348675	21678833	27726895	21993690
rRNA	19314195	21637189	27664792	21957108
**ASSEMBLY**				
Contigs	327777	346669	460995	337130
Singletons	2800174	2937361	3951554	2842044
% assembled reads	85,50%	86,42%	85,72%	87,06%
**TBEV RESULTS**				
Nb contigs	4	127	143	104
Nb reads in contigs	100031	2514666	2255875	2060105
Nb singletons	145	5052	3932	3 570
Total reads	100176	2519718	2259807	2 063 675
Average contigs identity (%)	91,23%	92,06%	91,71%	90,53%
**ADDITIONAL CLOSE SPECIES (TOTAL READS*)**				
Machupo mammarenavirus	1	2		
Louping ill virus		14		
Bovine viral diarrhea virus 1	12	7	9	9

Bovine viral diarrhea virus 2		1		
Bovine viral diarrhea virus 3		1		
Orthohepevirus A				1
Singapore grouper iridovirus	48		41	59
Rotavirus C	1			
Cercopithecine betaherpesvirus 5		25		2
Stealth virus 4				5
Stealth virus 5			1	
Human gammaherpescirus 8		23	6	
uncultured virus			30	
Simian retrovirus		3	17	
Squirrel monkey retrovirus	923291	837907	1064215	1 107 994
Primate T-lymphotropic virus 1	5	29	10	
Baboon endogenous virus	8503	8691	7710	10 617
Feline leukemia virus	2		3	28
Human endogenous retrovirus	478	280	529	322
Human endogenous retrovirus K	16	35	37	37
Human endogenous retrovirus W	64	53	37	60
Lnras*SN acutely transforming retrovirus				9
Retroviridae (no genus, no species)			4	7

* known false hits due to bad annotations in database have been removed
