# Supplementary material for: Adventitious Virus Detection in Cells by High-Throughput Sequencing of Newly Synthesized RNAs: Unambiguous Differentiation of Cell Infection from Carryover of Viral Nucleic Acids
Source: mSphere. 2019 Jun 5;4(3):e00298-19. doi: 10.1128/mSphere.00298-19 (PMC6553555; doi:10.1128/mSphere.00298-19)
Supplement: FIG S1 [file mSphere.00298-19-sf001.pdf]

```
SMRV-V 1    MGQASSHSENDLFISQLKESLKVRRIRVRKKDLVSFFSFIFKTCPWFPQEGSIDSRVWGR  60
            MGQASSHSENDLFIS LKESLKVRRIRVRKKDLVSFFSFIFKTCPWFPQEGSIDSRVWGR       p19
SMRV-H 1    MGQASSHSENDLFISHLKESLKVRRIRVRKKDLVSFFSFIFKTCPWFPQEGSIDSRVWGR  60

SMRV-V 61   VGDCLNDYYRVFGPETIPITTFNYYNLIRDVLTNQGDSPDIQRLCKEGHKILISHSRPPS  120
             VGDCLNDYYRVFGPETIPITTFNYYNLIRDVLTNQ DSPDIQRLCKEGHKILISHSRPPS
SMRV-H 61   VGDCLNDYYRVFGPETIPITTFNYYNLIRDVLTNQSDSPDIQRLCKEGHKILISHSRPPS  120

SMRV-V 121  RQAPVTITTSEKASSRPPSRAPSTCPSVAIDIGSDDTGQSSLYPNLATLTDPPIQSPHSR  180
            RQAPVTITTSEKASSRPPSRAPSTCPSVAIDIGS DTGQSSLYPNLATLTDPPIQSPHSR
SMRV-H 121  RQAPVTITTSEKASSRPPSRAPSTCPSVAIDIGSHDTGQSSLYPNLATLTDPPIQSPHSR  180

SMRV-V 181  AHTPPQHLPLLANSKTLHNSGSQDDQLNPADQADLEEAAAQYNNPDWPQLTNTPALPPFR  240
            AHTPPQHLPLLANSKTLHNSGSQDDQLNPADQADLEEAAAQYNNPDWPQLTNTPALPPFR        P16
SMRV-H 181  AHTPPQHLPLLANSKTLHNSGSQDDQLNPADQADLEEAAAQYNNPDWPQLTNTPALPPFR  240

SMRV-V 241  PPPYVPTAVPPVAVAAPVLHAPTSGVPGSPTAPNLPGVALAKPSGPIDETVSLLDGVKTL  300
            PP YV TAVPPVAVAAPVLHAPTSGVPGSPTAPNLPGVALAKPSGPIDETVSLLDGVKTL
SMRV-H 241  PPSYVSTAVPPVAVAAPVLHAPTSGVPGSPTAPNLPGVALAKPSGPIDETVSLLDGVKTL  300

SMRV-V 301  VTKLSDLALLPPAGVMAFPVTRSQGQVSSNTTGRASPHPDTHTIPEEEEADSGESDSEDE  360
            VTKLSDLALLPPAGVMAFPVTRSQGQVSSNTTGRASPHPDTHTIPEEEEADSGESDSED+
SMRV-H 301  VTKLSDLALLPPAGVMAFPVTRSQGQVSSNTTGRASPHPDTHTIPEEEEADSGESDSEDD  360

SMRV-V 361  EEESSEPTEPTYTHSYKRLNLKTIEKIKTAVANYGPTAPFTVALVESLSERWLTPSDWFF  420
            EEESSEPTEPTYTHSYKRLNLKTIEKIKTAVANYGPTAPFTVALVESLSERWLTPSDWFF
SMRV-H 361  EEESSEPTEPTYTHSYKRLNLKTIEKIKTAVANYGPTAPFTVALVESLSERWLTPSDWFF  420

SMRV-V 421  LSRAALSGGDNILWKSEYEDISKQFAERNARKASSKGWTLKKFLGASPYQNNDKQAQFPP  480
            LSRAALSGGDNILWKSEYEDISKQFAER   +   K   L K  GASPYQNNDKQAQFPP       p35
SMRV-H 421  LSRAALSGGDNILWKSEYEDISKQFAERTRVRPPPKDGPL-KIPGASPYQNNDKQAQFPP  479

SMRV-V 481  GLLTQIQSAGLKAWKRLPQKGAATTSLAKIRQGPDESYSDFVSRLQETADRLFGSGESES  540
```

```
           GLLTQIQSAGLKAWKRLPQKGAATTSLAKIRQGPDESYSDFVSRLQETADRLFGSGESES
SMRV-H 480  GLLTQIQSAGLKAWKRLPQKGAATTSLAKIRQGPDESYSDFVSRLQETADRLFGSGESES  539

SMRV-V 541  SFVKHLAYENANPACQSAIRPFRQKELCDYVRLCSGIGSAHAVGLAIGAALQNLAPAQLP  600
            SFVKHLAYENANPACQSAIRPFRQKEL     L     SAHAVGLAIGAALQNLAPAQL
SMRV-H 540  SFVKHLAYENANPACQSAIRPFRQKELSTMSPLL-WYCSAHAVGLAIGAALQNLAPAQLL  598

SMRV-V 601  GAQARLCYNCHQPGHLSRNCPQKIQPPTQLPTQPNAPQTSLIKNLGPTTKCPRCKKGFHW  660
              +         P       P+KIQPPTQLPTQPNAPQ SLIKNLGPTTKCPRCKKGFHW      p10
SMRV-H 599  EPRPAFAIIVTNPAIFQETAPKKIQPPTQLPTQPNAPQASLIKNLGPTTKCPRCKKGFHW  658

SMRV-V 661  ASECRSRLDINGQPIIRQGNLNRGQPQGPTTGMNSGASQFTPQYRQPTPALPVINHAATS  720
            ASECRSRLDINGQPII+QGNLNRGQPQGPTTGMNSGASQFTPQYRQPTPALPVINHAATS
SMRV-H 659  ASECRSRLDINGQPIIKQGNLNRGQPQGPTTGMNSGASQFTPQYRQPTPALPVINHAATS  718

SMRV-V 721  QTSGEQQRAVQDWTSVPPPTQY  742
            QTSGEQQRAVQDWTSVPPPTQY
SMRV-H 719  QTSGEQQRAVQDWTSVPPPTQY  740
```
